# Bladder cancer-related microbiota: examining differences in urine and tissue samples

**DOI:** 10.1038/s41598-020-67443-2

**Published:** 2020-07-06

**Authors:** Bassel Mansour, Ádám Monyók, Nóra Makra, Márió Gajdács, István Vadnay, Balázs Ligeti, János Juhász, Dóra Szabó, Eszter Ostorházi

**Affiliations:** 1 Department of Urology, Markhot Ferenc University Teaching Hospital, Eger, Hungary; 2 Department of Medical Microbiology, Semmelweis University, Nagyvárad tér 4, 1089 Budapest, Hungary; 3 Department of Pharmacodynamics and Biopharmacy, Faculty of Pharmacy, University of Szeged, Szeged, Hungary; 4 Department of Pathology, Markhot Ferenc University Teaching Hospital, Eger, Hungary; 5 Faculty of Information Technology and Bionics, Pázmány Péter Catholic University, Budapest, Hungary

**Keywords:** Cancer, Microbiology, Molecular biology, Oncology, Urology

## Abstract

The microbiota isolated from the urine of bladder carcinoma patients exhibits significantly increased compositional abundance of some bacterial genera compared to the urine of healthy patients. Our aim was to compare the microbiota composition of cancerous tissues and urine samples collected from the same set of patients in order to improve the accuracy of diagnostic measures. Tissue samples were collected from patients during cancer tissue removal by transurethral resection. In parallel, urine samples were obtained by transurethral resectoscopy from the same patients. The V3–V4 region of the bacterial 16S rRNA gene was sequenced and analyzed using the Kraken pipeline. In the case of four patients, duplicate microbiota analysis from distant parts of the cancerous tissues was highly reproducible, and independent of the site of tissue collection of any given patient. *Akkermansia, Bacteroides, Clostridium *sensu stricto*, Enterobacter* and *Klebsiella,* as “five suspect genera”, were over-represented in tissue samples compared to the urine. To our knowledge, this is the first study comparing urinary and bladder mucosa-associated microbiota profiles in bladder cancer patients. More accurate characterization of changes in microbiota composition during bladder cancer progression could provide new opportunities in the development of appropriate screening or monitoring methods.

## Introduction

Bladder cancer ranks the ninth most frequently-diagnosed cancer worldwide with 75% cases occurring in men^1^. While the main risk factor for bladder cancer is tobacco smoking^2^, exposure to aromatic amines^3^ or environmental factors such as arsenic in drinking water^4^ have been classified as additional carcinogenic risks. In addition, genetic factors including slow acetylation of *N*-acetyltransferase, a key enzyme in aromatic amines metabolism, are believed to play roles in bladder cancer development^5^.

Urine was traditionally considered sterile; however recent evidence has challenged this dogma by molecular-based detection of microorganisms in urine of healthy individuals^6^. Although the presence of microbes in the urinary tract does not necessarily induce infections, some microbial agents do cause acute infection or chronic inflammation. On the same token, certain commensal strains may control the overgrowth of pathogenic bacterial strains. The association between bladder schistosomiasis infection, inflammation and squamous cell carcinoma is well accepted^7^, but the role of bacteria in the pathophysiology or management of bladder cancer has not been consistently examined. In addition to the genetic characteristics that influence the elimination of chemical carcinogens, the processes can be aggravated or attenuated by the presence of biochemically active microbes. For example, nitrate producing bacteria can mediate the formation of carcinogenic *N*-nitrosamines^8^. Toxins including heavy metals, pesticides, ochratoxins, polycyclic aromatic hydrocarbons or other environmental contaminants are removed from the bloodstream through renal filtration. All these compounds interact with the microbiota during ensuing storage in the bladder. The resulting metabolites can increase or decrease the risk of bladder cancer.

Several factors can influence the difference in incidence and progress of bladder cancer between men and women. The study of de Jong et al. described that male hormones may influence the type of bladder cancer that a patient develops^9^. The urinary microbiota is different in men and women^6^. The anatomical and hormonal differences lead not only to higher incidence of female urinary tract infections, but to different compositions of urinary microbiota between genders. Incidence of female bladder cancer is significantly lower compared to bladder oncogenesis of men. The different microbiota can be one of the hypotheses for the lower incidence of bladder cancers in women^10,11^.

The urinary microbiota associated with benign urologic conditions from transurethral catheterized urine or from voided urine was reviewed in detail^10^. In women voided urine samples contained mixtures of urinary and genital tract bacteria, however the uncultivated bacteria, assessed by 16S rRNA gene sequencing were common in voided, transurethral catheterized or suprapubic aspirated urine samples^12^. In men patients with or without lower urinary tract symptoms voided urine does not adequately characterize the male bladder microbiota, and only the detectable bacteria in catheterized urine were associated to the severity of symptoms^13^.

Current knowledge of the microbiota in voided urine or tissue samples of bladder cancer patients is very limited^14–17^. As far as we know, no studies to date have compared the characteristics of catheterized urine with tissue samples in bladder cancer.

Our aim was to determine whether the microbiota composition in samples from different sites of a given tumor tissue is consistent and to compare the microbiota isolated from the tumor tissue with that from the urine of the same patients.

## Results

Using conventional culture methods, no bacteria were detected from the urine of any patient.

From the 39 urine and tissue paired samples collected, 24 samples provided sufficient DNA to meet the sequencing quality criteria. The 24 samples analyzed included 10 urine and 14 tissue samples collected from ten bladder cancer patients. Based on histological examination, four patients had muscle invasive (MIBC) and six patients had non-muscle-invasive (NMIBC) tumors. The male/female ratio was 2:4 in the NMIBC group, and 3:1 in the MIBC group (Table 1).

**Table 1:** Metadata of patients: MIBC: muscle invasive bladder cancer, NMIBC: non muscle invasive bladder cancer.

Patients ID	Age	Gender	Biological characteristic	Urine sample ID	Tissue sample ID	
I01	70	Male	MIBC	UI1	TI01.1	
					TI01.2	
I02	80	Male	MIBC	UI2	TI02	
I03	79	Male	MIBC	UI3	TI03	
I04	76	Female	MIBC	UI4	TI04	
N01	72	Male	NMIBC	UN1	TN01.1	
					TN01.2	
N02	54	Male	NMIBC	UN2	TN02.1	
					TN02.2	
N03	66	Female	NMIBC	UN3	TN03.1	
					TN03.2	
N04	58	Female	NMIBC	UN4	TN04	
N05	20	Female	NMIBC	UN5	TN05	
N06	64	Female	NMIBC	UN6	TN06	
Summary	63.9	Male/female = 5/5	MIBC/NMIBC = 4/6	10	14	24

A total of 3.2 million valid sequences were obtained, resulting in 1.2 million high-quality reads. Within one sample, the median number of reads was 119,268.

**Figure 1:**
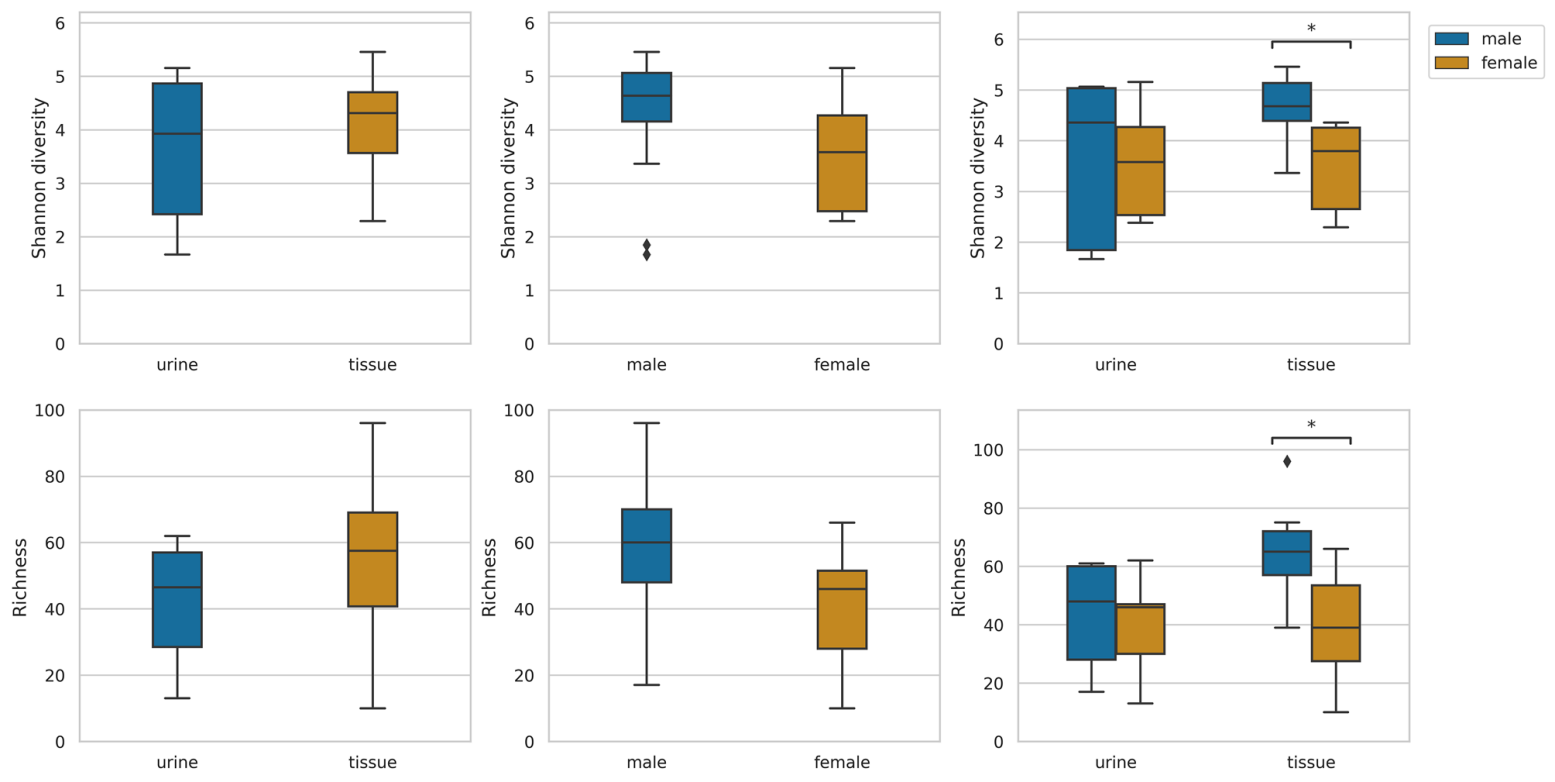
Observed richness and Shannon diversity at genus level from the urine and tissue samples obtained from patients: significant difference was shown only between the median of genus richness and Shannon diversity of female and male tissue samples.

Figure 1 shows the observed richness and Shannon diversity at the genus level, comparing the gender and sample size groups. Statistically significant differences were found only between the median of genus richness and Shannon diversity representing female and male tissue samples. Shannon diversity of female patients’ transurethral catheterized urine samples were lower than that of male patients (not significant).

**Figure 2:**
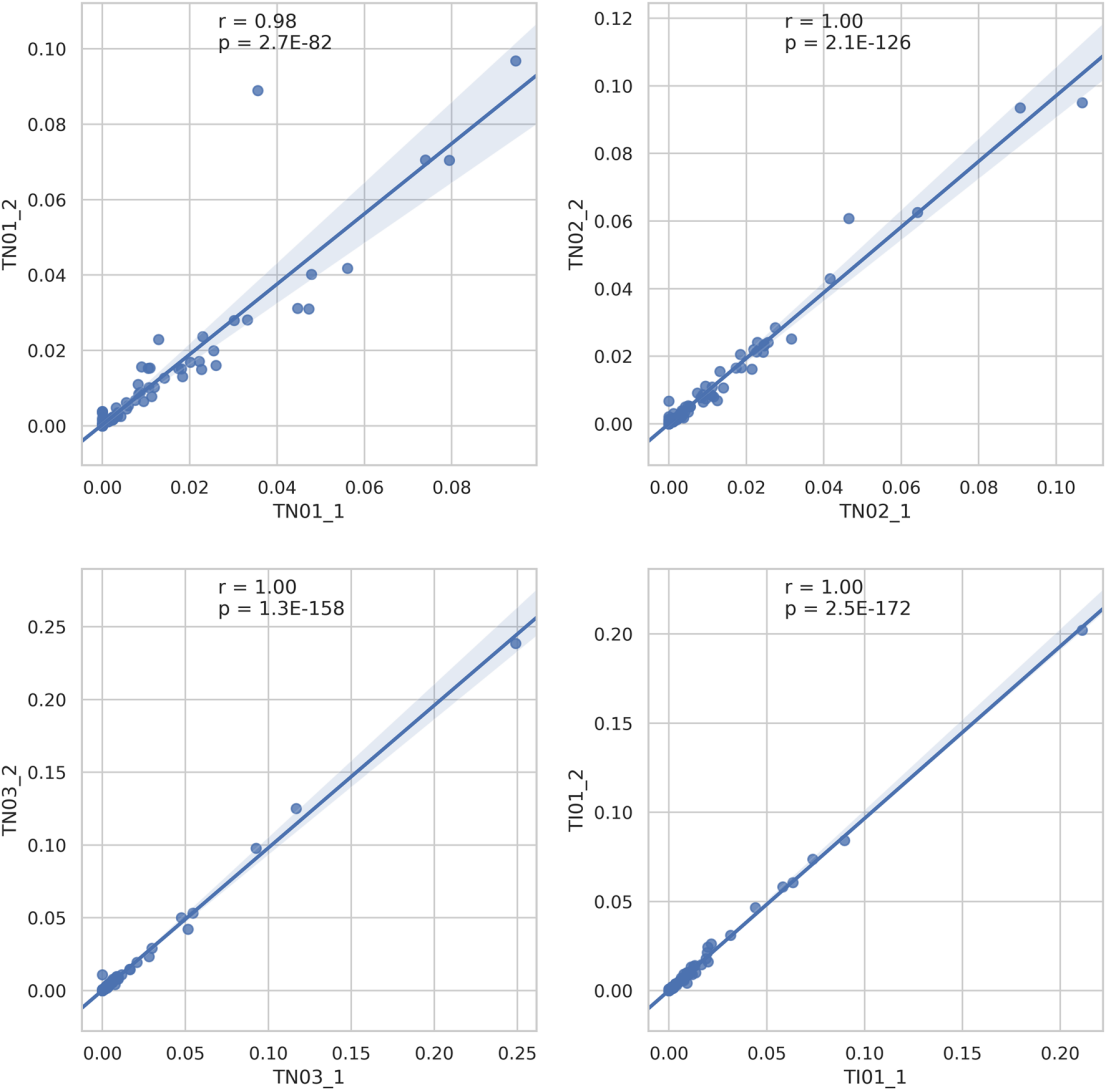
Correlation-based reproducibility of the microbiota from bladder cancer tissue: Scatter plots A, B, C and D show the abundance of genera taken from site 1 and site 2 of a subject (TN01, TN02, TN03, TI01). Pearson correlations were calculated for all the 4 subjects. All correlations show strong statistical significance.

**Figure 3:**
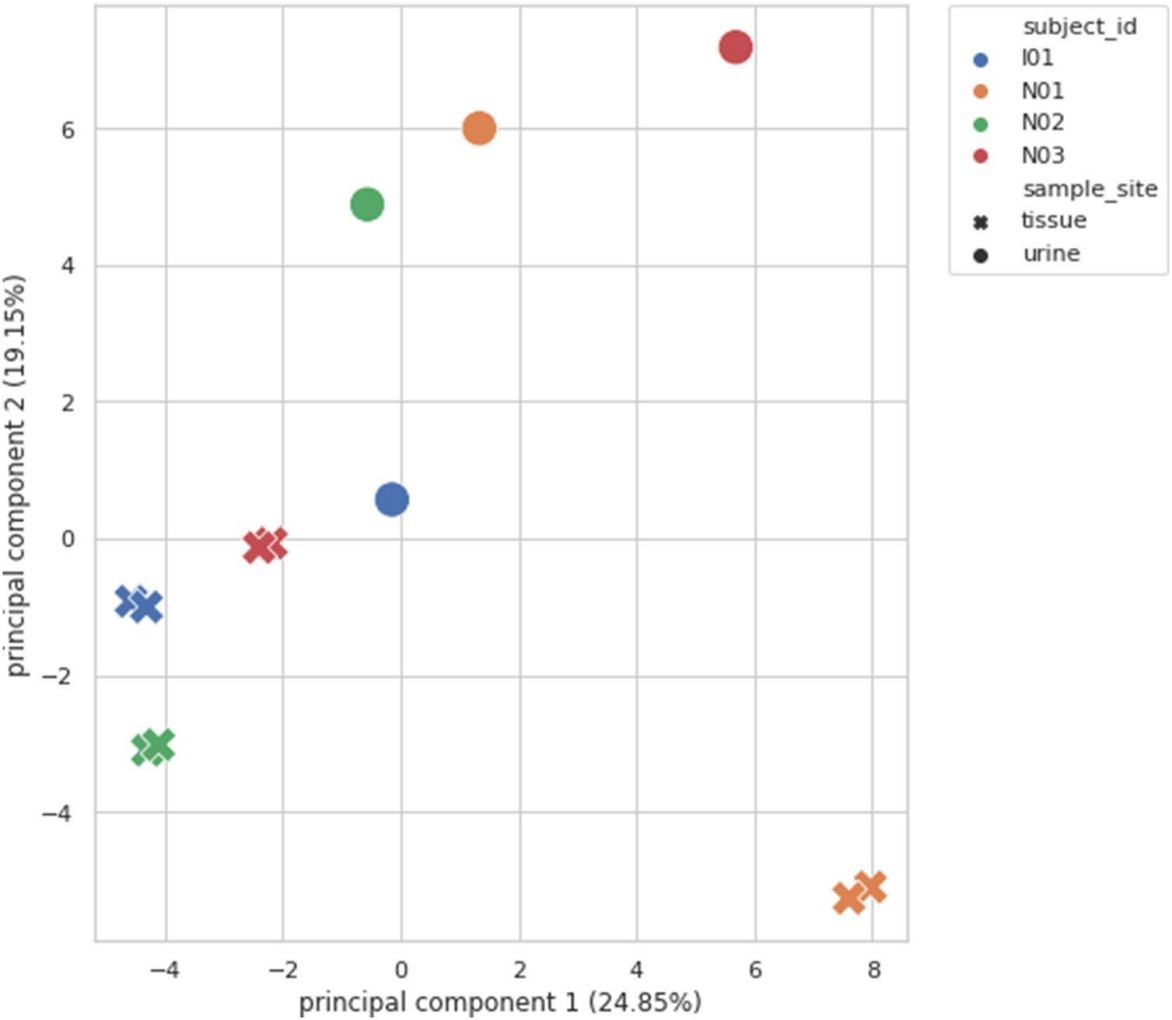
Principal component analysis (PCA) of correlated bladder cancer tissue samples with their respective urine samples: correlated tissue samples did not cluster with their respective urine samples.

**Figure 4:**
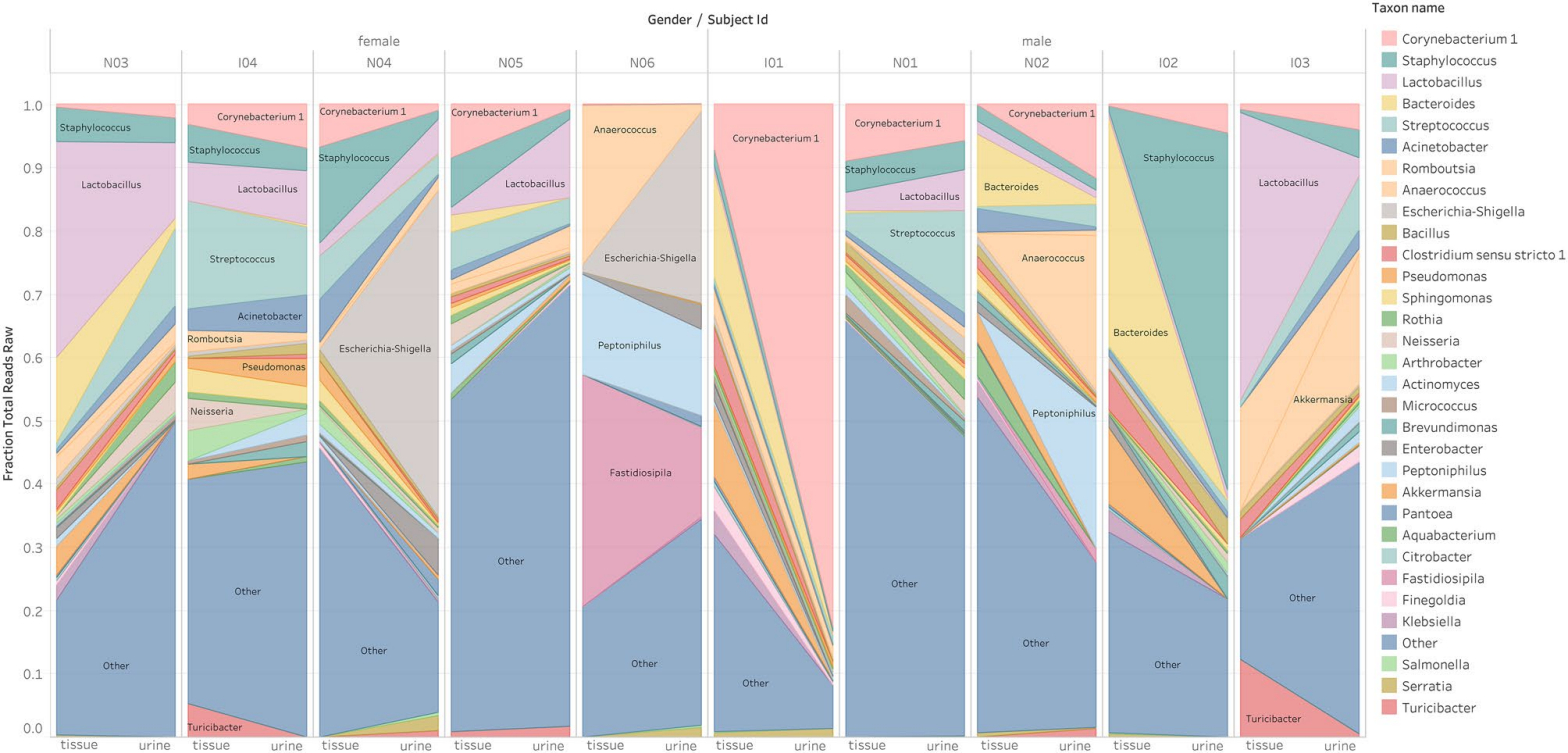
Distribution of genera in the microbiota of the urine and tissue samples obtained from the same patients. Each bar represents the microbiota results from the indicated patient, the most abundant genera are shown on the left and right sides from tissue and urine samples, respectively.

From two isolated distant pieces of the tumor, microbiota analysis was performed in four patients. Figure 2, correlation-based reproducibility measurements of bladder cancer tissue microbiota demonstrate that the microbiota analysis results were reproducible, independent of the actual site and highly specific to any given person. Pearson correlation coefficients were 0.98–1, and p values were < 0.05. However, at the Principal Component Analysis (PCA), Fig. 3 the correlated tissue samples do not cluster with their own urine samples. No regularity or tendency was shown regarding dominant microorganisms, there was no rule for how the abundance of genus changes between tissue and urine (Fig. 4).

**Figure 5:**
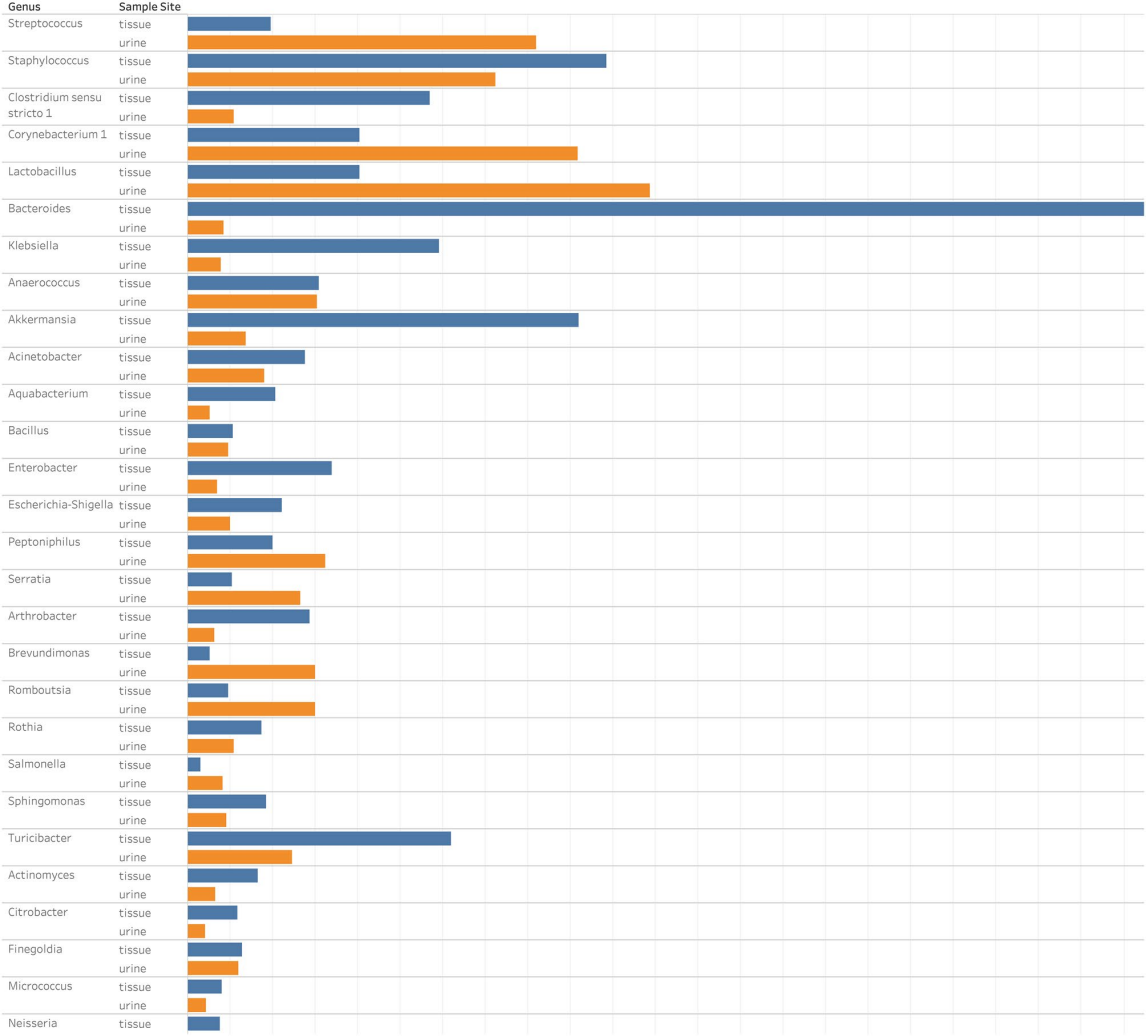
Comparative analysis of median fraction total reads at level of genera detected from the microbiota of the urine and tissue samples obtained from patients.

In urine samples, the most abundant phyla detected were *Firmicutes* with abundance of 33%, followed by *Proteobacteria* (29%), *Actinobacteria* (23%), *Cyanobacteria* (7%) and *Bacteroidetes* (4%). In the tissue samples, the order was as follows: *Firmicutes* (34%), *Actinobacteria* (23%), *Proteobacteria* (22%), *Bacteroidetes* (15%) and *Cyanobacteria* (8%). The most abundant genera in urine samples taken as a whole were *Lactobacillus, Corynebacterium*, *Streptococcus* and *Staphylococcus*, with age and gender differences in each samples (Table 2). In tissue samples the most abundant genera were *Bacteroides*, *Akkermansia, Klebsiella* and *Clostridium *sensu stricto. *Akkermansia, Bacteroides, Clostridium *sensu stricto*, Enterobacter* and *Klebsiella* genera showed remarkably higher compositional abundance in tissue than in urine samples (Fig. 5).

**Table 2:** The most abundant bacterial genera in urine samples by ages and gender of patients.

Age group	Female	Male
20–49 years	**Akkermansia** Atopobium **Bifidobacterium** Gardnerella **Gemella** Lactobacillus **Romboutsia** Staphylococcus Streptococcus **Turicibacter**	**No patient in the study**
50–69 years	**Actinotignum** Enterobacter **Escherichia-Shigella** Fastidiosipilia Lactobacillus **Neisseria** **Parvimonas** Peptoniphilus Streptococcus **Williamsia**	Anaerococcus Corynebacterium **Fastidiosipila** **Lautropia** Peptoniphilus Porphyromonas Prevotella Staphylococcus **Streptococcus** **Varibaculum**
70 + years	**Acinetobacter** Actinomyces Corynebacterium Fusobacterium Lactobacillus **Massilia** **Pseudomonas** **Sphingomonas** Staphylococcus Streptococcus	Anaerococcus **Bacillus** **Brevundimonas** Corynebacterium **Deinococcus** Lactobacillus **Peptoniphillus** **Rubellimicrobium** Staphylococcus **Streptococcus**

Those highlighted in bold are not members of CORE bacteria in healthy population according to Lewis et al.^11^.

**Figure 6:**
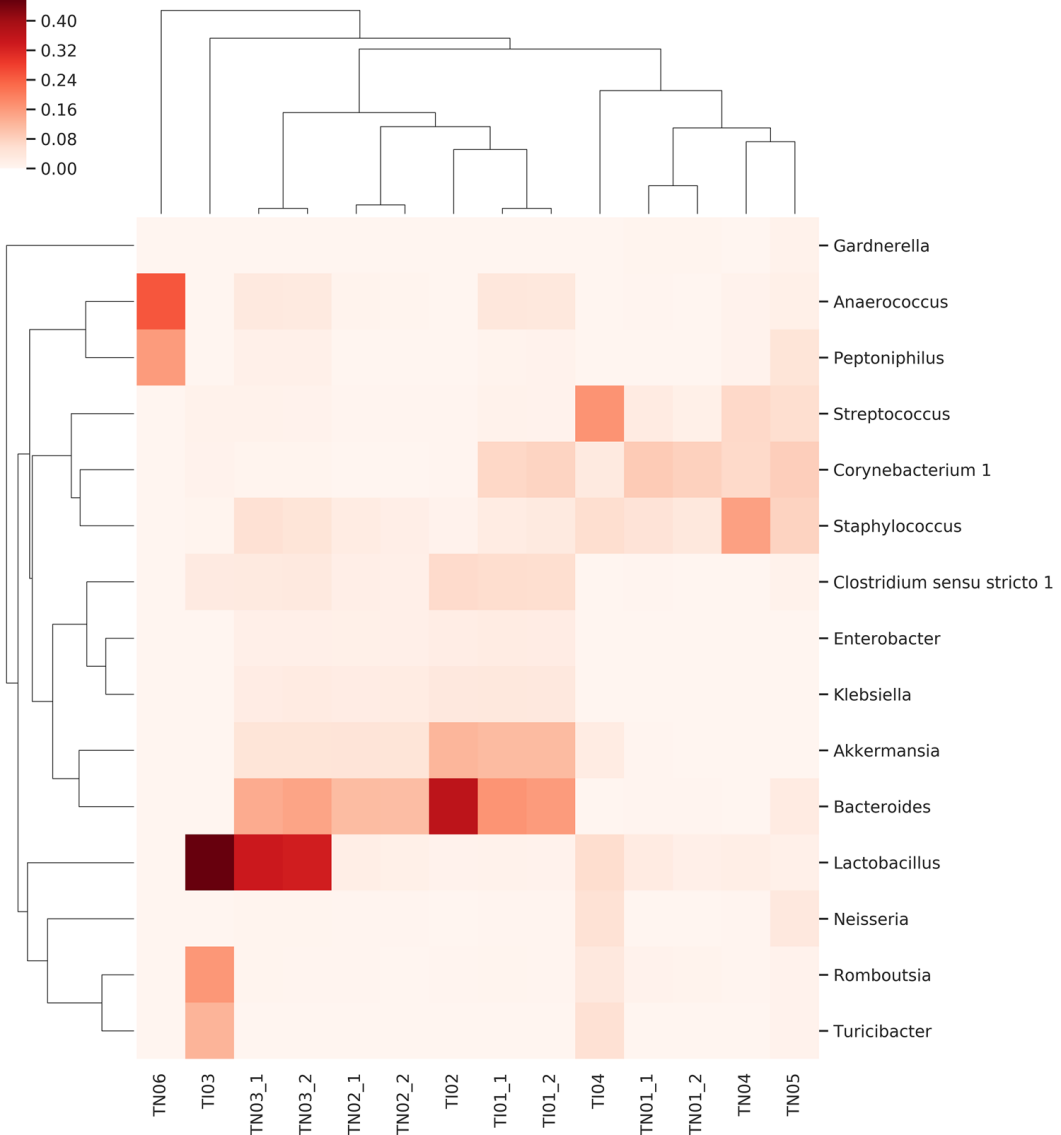
Heat-map of the distribution of co-existing genera in cancer tissue samples obtained from NMIBC (with TN designation) and MIBC (with TI designation) patients. Evidence of a correlation between the biological characteristics of the tumor and the distribution of the bacteria is not confirmed. Regardless of the biological characteristics of the tumor the co-existence of 5 genera (namely *Clostridium *sensu stricto, *Akkermansia*, *Bacteroides*, *Enterobacter* and *Klebsiella*) in tissue samples is characteristic.

The Fig. 6 heat map shows the coexistence of five genera in tissue samples. *Clostridium *sensu stricto*, Akkermansia, Bacteroides, Enterobacter* and *Klebsiella* are characteristic but not general for all samples. No relationship was found between the biological characteristic of cancer [muscle invasive bladder cancer (MIBC) versus non-muscle invasive bladder cancer (NMIBC)] and microbiota composition.

## Discussion

Since there were no significant differences between the richness and Shannon diversity values of the transurethral catheterized urine and tissue microbiota, we might even lead to think that a urine microbiota test is suitable as a diagnostic and prognostic tool in bladder tumor research. However, the fact that there are significant differences between the diversity of female and male tissue samples confirms that transurethral catheterized urine samples are not mirror images of the microbiota at the site of the cancer. Clearly, the role of tissue associated bacteria in tumor progression has to be addressed in future studies.

In agreement with the results of Bucevic et al.^15^, *Firmicutes* was the most common phylum in both our tissue and urine samples. Two Chinese study groups^16,17^ identified *Proteobacteria* as the most abundant phylum in urine and tissue samples of bladder cancer patients. While none of the earlier reports document *Cyanobacteria* as a significant phylum, in our urine and tissue samples its abundance was 7 and 8%, respectively. Microcystins are toxic products of *Cyanobacteria* that induce hepatocellular cancer and promote migration and invasion of colorectal cancer^18,19^. Assuming that a change in the gut microbiota also results in a change of the bladder microbiota, different geographical locations and dietary patterns may play a role in the increased presence of *Cyanobacterium* in our samples^20,21^. Further studies are required to quantify the difference in the amount of microcystin-producing species in the urine of bladder cancer patients and controls. Provided that microcystins also play a role in the development of bladder cancer, we should consider the individual genetic differences in the microcystin detoxification process between cancerous and healthy people^22^.

Patients with different urinary tract diseases—neurogenic bladder dysfunction, urgency urinary incontinence or interstitial cystitis—reflect altered urinary tract microbiota in contrast to healthy volunteers^23–25^. Nevertheless, the urinary microbiota of a healthy population is ambiguous, because healthy control participants in the relevant studies belonged to different age groups and different genders. Lewis et al.^6^ propose the existence of a core urinary microbiota—a subset of bacteria present at variable abundances within the clean-catch, mid-stream voided urine—regardless of age and gender. In the age group of 20–49 years our study was limited to one female participant with *Gardnerella, Lactobacillus* and *Streptococcus* as the most abundant genera in her urine sample. These bacteria belong to the core urinary microbiota according to the Lewis classification. In the urine samples of our female age group 50–69 years, in addition to the *Peptinophilus, Parvimonas, Streptococcus, Lactobacillus* and *Fastidiosipila* genera belonging to the core, most frequent bacteria observed were *Escherichia-Shigella, Actinotignum* and *Williamsia*. In the 76 years old female patient (age group 70 +) the most abundant genera, namely *Streptococcus, Lactobacillus* and *Corynebacterium*, were all members of the core. The most common genera in urine of our male patients, regardless of age, was *Anaerococcus, Corynebacterium, Peptoniphilus*, and *Staphylococcus,* genera belonging to the core and *Streptococcus* outside of the core.

When compared the urine of 6 healthy individuals with 8 patients of urothelial cancer, Xu et al.^14^, observed the enrichment of *Streptococcus* in the urine from patients with urothelial carcinoma. In contrast, when Bucevic et al.^15^ analyzed 11 healthy and 12 patients with bladder cancer, the most abundant genera in healthy patients’ urine were *Streptococcus, Veillonella* and *Corynebacterium*, however *Fusobacterium, Actinobaculum, Facklamia* and *Campylobacter* genera enrichment representing the urine of bladder cancer patients. In the urine of our patients *Streptococcus, Corynebacterium* and *Fusobacterium* genera were similarly enriched. Wu et al.^16^ found significantly higher relative levels of *Acinetobacter, Anaerococcus, Rubrobacter, Sphingobacterium, Atoposites* and *Geobacillus* in urine of cancer patients. While in our patients the abundances of *Acinetobacter* and *Anaerococcus* increased depending on the age and gender group, the other four genera occurred only in minor quantities. Inter-individual variability was observed between urinary microbiotas of the participants in our study. Having said that, the most abundant genera in our samples were those detected by earlier reports. When differences were found, the reason for the alterations may be the low number of samples, ethnicity, gender and/or age. In addition, we collected the urine directly from the bladder by resectoscopy and not by the clean-catch voided urine method.

Inter-individual variability was observed among the microbiota of tissue samples. However, 5 genera, *Akkermansia, Bacteroides, Clostridium *sensu stricto*, Enterobacter* and *Klebsiella* were over-represented in tissue samples compared to the urine. This difference indicates that these genera are directly associated with the tissue and further studies with larger sample sizes of cancerous bladder mucosa and adjacent normal healthy tissues are needed to clarify the genera associated with the oncogenic transformation process. For now, it is impossible to determine whether the high abundance of these genera is the cause or consequence of tumorigenesis. The link between chronic inflammation, microbiota and the progression of solid tumors has been established especially for colorectal cancer (CRC). In the CRC animal experiments of Baxter et al.^26^, the taxa *Bacteroides* and *Akkermansia* were strongly positively correlated with an increased tumor burden, but *Clostridium* was associated with a decreased rate of malignant transformation. *Bacteroides* and *Akkermansia* both are known mucin degraders, they undermine the integrity of the mucosal barrier, leading to increased inflammation^27,28^. The different species from *Clostridium* genera play different roles in carcinogenesis. The butyrate-producing *Clostridium spp.* inhibits intestinal tumor development^29^ as much as *Clostridium septicum* does not appear to initiate carcinogenesis but appears to have a symbiotic relationship with the growth of already developing malignancies^30^. The study of Yurdakul et al. shows that *Enterobacter *spp. may be a clinically important factor for colon cancer initiation and progression due to apoptosis inhibition^31^. The colibactin toxin produced by *Klebsiella pneumonia* is known to cause DNA double-strand breaks inducing genomic instability as well as cell cycle arrest^32^. The continuous insult of colibactin toxin is supplemented with the influx of proinflammatory cytokines into the colonic microenvironment in the presence of *Klebsiella*, leads to chronic inflammation, and stimulation of epithelial cell proliferation^33^. The strong correlation between the appearance of these “five suspect genera” in our samples suggests that this set of bacteria mutually enhances each other's carcinogenic effects. Fusobacterial DNA has been detected in pancreatic, esophageal or colorectal cancers^34,35^. Bucevic et al.^15^ detected *Fusobacterium* in 25% of urine samples of bladder cancer patients. None of our tissue samples contained *Fusobacterium* DNA at the level of detection, but it was represented in all our urine samples. Liu et al. found a higher abundance of *Cupriavidus, Acinetobacter, Anoxybacillus, Escherichia–Shigella, Geobacillus, Pelomonas, Ralstonia* and *Sphingomonas* genera in the cancerous relative to healthy tissues of the same bladder cancer patients^17^. In our study *Cupriavidus* and *Pelomonas* were represented only in tissue samples; the urine of these two patients did not contain any detectable amount of these genera. *Acinetobacter, Anoxybacillus, Escherichia-Shigella, Geobacillus, Ralstonia* and *Sphingomonas* were increasingly represented in our tissue samples compared to the urine samples. The fact that these latter genera, and our “five suspects” are detectable in greater quantities in cancerous tissue than in the urine supports the hypothesis that these bacteria were associated with the tumor-altered tissue.

The small number of patients (5 male and 5 female) is a limitation of this study, and restricts the conclusions that may be drawn on the impact of gender differences on microbiota changes in bladder cancer development. Additional, more exhaustive studies are required to obtain increasingly accurate data for the better understanding of cause-effect relationships between microbiota and bladder cancer.

Tumor tissue size limited the ability to perform microbiota analysis in duplicate from each tissue sample.

Using the 16S rRNA sequencing method, analyzed by the Kraken-Bracken pipeline is not validated to infer taxonomic information below the genus level, therefore future studies may harness species-level analysis to identify specific microbes which may have associations with bladder cancer.

Limitation of the study is that without using samples of healthy controls—due to obvious ethical reasons—it is not yet clear whether the „five suspect bacterial genus” are associated exclusively with bladder cancer tissue.

However, this is the first report that evaluates urine samples directly from the bladder, avoiding urethra contamination. Moreover, our study suggests that the bladder-mucosa associated microbiota is different from the urinary microbiota and provides the best test sample to characterize the association between bacteria and carcinogenesis.

Herein, we profiled the bladder mucosa microbiota compared to the urinary microbiota associated with bladder cancer in the most comprehensive study to date. Our study reflected that urine samples provide different results relative to tissue samples during the characterization of the bacteria involved in carcinogenesis. An extension and deepening of these investigations lead to a better understanding of the pathogenesis of bladder cancer, and will provide novel diagnostic and therapeutic options.

## Methods

### Ethics approval and consent to participate

Sample collection protocols were approved by the Ethics Committee of Markhot Ferenc University Teaching Hospital (MFUTH) and the Ethics Committee of Semmelweis University (SE RKEB: 100/2018). The present study was conducted in accordance with ethical standards that promote and ensure respect and integrity for all human subjects and the Declaration of Helsinki. Patients treated at the MFUTH's Urology Department between May and October 2018 were enrolled in this study, all research was performed in accordance with guideline and regulation of MFUTH, and written informed consent was obtained from all patients.

### Sample collection

The study included five male and five female patients with bladder carcinoma. Exclusion criteria were antibiotic or probiotic treatment 2 weeks prior to surgery or recent urinary infection. The patients’ clinical parameters, and the metadata of the samples are presented in Table 1. Mucosal tissue samples removed during transurethral resection (TUR) were divided to histological and microbiota analysis. Urine samples were collected directly from the bladder during the TUR surgery and divided for traditional routine culture and microbiota analysis. Routine urine culture involves inoculation of 10 µl of urine on to blood and eosin-methylene blue agar plates, and 24-h incubation under ambient atmospheric conditions. The level of detection is 100 colony-forming units per milliliter (CFU/ml). Fractions for microbiota analysis were centrifuged at 16,000 *g* for 10 min, supernatants were aliquoted and stored at − 80 °C.

### DNA isolation, 16S rRNA gene library preparation and MiSeq sequencing

DNA isolation was performed by ZymoBIOMICS DNA Miniprep Kit (Zymo Research Corp., Irvine, USA) according to manufacturer’s instructions after enzymatic dissolution with ProtK (56 °C, 3 h) for the tissue samples. Enzymatic digestion was performed with standardized 2 mm in diameter tissue pieces.

Concentration of genomic DNA was measured using a Qubit2.0 Fluorometer with Qubit dsDNA HS Assay Kit (Thermo Fisher Scientific, Waltham, MA, USA). Bacterial DNA was amplified with tagged primers covering the V3–V4 region of bacterial 16S rRNA gene. PCR and DNA purifications were performed according to Illumina’s protocol. PCR product libraries were assessed using DNA 1000 Kit with Agilent 2100 Bioanalyzer (Agilent Technologies, Waldbronn, Germany). Equimolar concentrations of libraries were pooled and sequenced on an Illumina MiSeq platform (Illumina, San Diego, CA, USA) using MiSeq Reagent Kit v3 (600 cycles PE).

In order to evaluate contribution of extraneous DNA from reagents, extraction negative controls and PCR negative controls were included in every run. To ensure reproducibility, each urine sample was independently extracted and sequenced twice. The full tissue samples of four patients were larger than 10 mm in diameter. These samples were cut into small pieces, two pieces originally located in a distance were separately digested enzymatically. All ensuing analytical steps, including the isolation of DNA, PCR, library preparation, sequencing and analysis procedures were done in duplicate.

Isolated DNA samples were placed at − 80 °C until PCR amplification.

Raw sequencing data were retrieved from the Illumina BaseSpace. Quality control (QC) of raw reads was carried out by FastQC and MultiQC^36^. After QC, low quality reads were trimmed by Trimmomatic^37^. The first 12 base pairs and consecutive low base calls were removed (phred score < 20) and only reads with minimal length of 36 were kept. The SSU Ref NR 99 database, downloaded on 22/03/2019 (release 132) of SILVA was used^38^. This contains non-redundant sequences (identity threshold: 99%). The database fasta file was pre-processed and indexed by the Kraken2^39^ tool (kraken2-build) with k-mer = 31. Kraken2 aligned and classified short-reads and the final estimation of taxon abundances in various taxonomic levels was performed with Bracken^40^.

Richness (number of unique taxa in the sample) and Shannon index (quantifying entropy of the distribution of taxa proportions) were used to quantify alpha diversities in microbiotas. Only those taxa were considered to be positive that had at least support of 50 reads; all others were discarded. Data were processed in python 3.7 with scikit-bio version 0.5.5. Bray–Curtis distance measure was used for assessing the beta-diversities of the microbiota and principal component analysis (PCA) for visualizing microbiotas. Differential abundance testing was done using Wilcoxon rank-sum test (in tissue-urine pairs) and PERMANOVA^41^ was used to determine compositional similarities.

## Data Availability

The datasets generated during the current study are available in the Short Read Archive (SRA) under accession number: PRJNA604455.
